# Driving Behaviors 2–3 Years After Traumatic Brain Injury Rehabilitation: A Multicenter Case-Control Study

**DOI:** 10.3389/fneur.2019.00144

**Published:** 2019-03-07

**Authors:** Michelle McKerral, Alexander Moreno, Patricia Delhomme, Isabelle Gélinas

**Affiliations:** ^1^Center for Interdisciplinary Research in Rehabilitation of Greater Montreal (CRIR), CIUSSS du Centre-Sud-de-l'Île-de-Montréal, Montreal, QC, Canada; ^2^Departement of Psychology, Université de Montréal, Montreal, QC, Canada; ^3^French Institute of Science and Technology for Transport, Development and Networks (IFSTTAR), Versailles, France; ^4^Centre for Interdisciplinary Research in Rehabilitation of Greater Montreal (CRIR) - CISSS de Laval, and School of Physical and Occupational Therapy, McGill University, Montreal, QC, Canada

**Keywords:** automobile driving, driving behaviors, interdisciplinary neurorehabilitation, road accidents, social participation, traffic offenses, traumatic brain injury

## Abstract

**Introduction:** Driving an automobile is an important activity for the social participation of individuals with traumatic brain injury (TBI). Return to safe driving is usually addressed during rehabilitation, but we know little about driving behaviors in the years following TBI rehabilitation.

**Objective:** To explore self-reported and objective (official driving records) post-rehabilitation driving behaviors and offenses in individuals with TBI: (a) having passed a driving evaluation, (b) who did not undergo a driving evaluation, and (c) non-injured controls.

**Methods:** Cross-sectional design with 162 adults: (a) 48 participants with mild, moderate, or severe TBI whose drivers' license was suspended and reinstated following a driving evaluation during rehabilitation (TBI-DE; *M* = 42.2 years of age, *SD* = 11.5); (b) 24 participants with TBI who maintained their driving privileges without undergoing a driving evaluation (TBI-NE; *M* = 36.5 years of age, *SD* = 9.9); (c) 90 non-injured controls (*M* = 43.8 years of age, *SD* = 11.4). Participants with TBI were recruited from seven rehabilitation centers, 2–3 years after the end of rehabilitation in the province of Quebec, Canada. During a telephone interview, data were obtained regarding self-reported driving: (a) habits; (b) self-efficacy; (c) anger expression; (d) sensation-seeking; (e) violations/errors; (f) accidents, driving offenses, and demerit points for the two-year interval predating the study. Objective data for driving offenses, accidents, and demerit points were obtained from the automobile regulatory body for the same period and for the two-year interval before the injury for the TBI groups.

**Results:** Compared to non-injured controls, the TBI-DE group reported significantly lower scores for self-reported verbal aggressive expression of anger and driving violations/errors. Conversely, their official driving records showed significantly more demerit points for the last 2 years, and a significantly higher frequency of serious post-rehabilitation accidents (10), compared to the TBI-NE group (one) and the control group (none). Compared to pre-injury levels, individuals with TBI had significantly more demerit points post-rehabilitation.

**Conclusions:** Individuals with TBI may underestimate risky driving behaviors even if they have been deemed fit to drive. Reduced self-awareness, memory, and dysexecutive problems following TBI could influence self-report of driving behaviors and explain discrepancies between self-reported and objective driving-related behaviors. Recommendations for research and practice are provided.

## Introduction

Traumatic brain injury (TBI) is a worldwide health problem resulting in long-lasting disability and negative psychosocial consequences, even in individuals with milder injuries (1). Successful return to driving following TBI has been positively associated with return to employment, life satisfaction, maintenance of social relationships, engagement in recreational activities, and community integration and participation (2). As the preferred mode of transport in the western industrialized world (3), driving a vehicle can also be a risky activity for the driver and for the society, with post-crash incapacity varying from 2 to 87% (4). The variability of published post-crash disability rates can be mainly explained by the specific outcome measures used and the modes of data collection and source (e.g., insurance claimants, hospital admissions, community settings). Between 42 and 85.1% of individuals with moderate to severe TBI return to driving and estimations indicate that 63% have not been professionally evaluated for driving competency (5–10). For individuals with severe TBI, a relicensing rate of 50% has been documented following extensive neurorehabilitation (11). Both stricter legal regulations (i.e., society's safety) and the individual with TBI's need to resume driving (i.e., personal safety and autonomy) are reasons for professionals to understand post-TBI driving behaviors and to develop reliable tools for their assessment (12).

Regarding the time necessary to return to driving, the results of a multicenter study indicated that 42% of individuals with TBI had returned to driving 1 year post-injury and the percentage increased to 53% at 5 years post-TBI (13). Even when individuals with milder injuries returned to driving faster, after 5 years, the severity of TBI was not a factor. Compared to pre-injury levels, estimates indicate that individuals with TBI changed their driving behaviors post-injury, including driving less frequently (e.g., 92.5% of drivers reported driving nearly every day pre-injury compared to 78.3% post-injury), driving more slowly (40.6%), limiting their driving times (36.8%), experiencing greater difficulties planning and remembering routes (41.5%), driving with fewer passengers (16%), avoiding night driving (24.5%), avoiding busy traffic (37.7%), and unfamiliar areas (19.8%), and more near-crashes (20%) (14, 15).

Driving can be an automatic and over-learned activity for experienced drivers, but it is far from being a routine activity because cognitive functions are necessary to effectively respond to changing environments and the continuing flood of complex information (12, 16). Cognitive deficits associated with TBI (17) may prevent individuals with TBI from driving safely, and compromise the driver's safety and that of other road users (18). Slowed reaction times (19), attentional problems (20, 21), anosognosia (22, 23), visuospatial problems (24–26), and dysexecutive symptoms (including behavioral and emotional control) (27, 28) have been associated with reduced fitness to drive (29). Neuropsychological assessment and on-road driving evaluations have been widely used to estimate the ability to resume driving following TBI (30, 31).

However, there are relatively few studies investigating driving behaviors in individuals who have returned to driving following a TBI with discordant findings. For example, the results of a study aiming to determine the frequency of road traffic accidents in 60 adults following severe TBI indicated that although 50% resumed driving, 63% of them were involved in traffic accidents with personal responsibility in 26/36 accidents (32). The authors concluded that compared to pre-injury levels, individuals with severe TBI who resume driving presented twice the risk of causing a road traffic accident. Also, in a study with a sample of 90 family caregivers of individuals with severe TBI, 32% of their care recipients had resumed driving but 38% of them had been involved in road traffic accidents (33). Compared to normative data, another study reported that the accident rate in individuals with TBI was more than two times higher (34). Furthermore, it was shown that individuals with acute mild TBI as well as individuals with TBIs of varying severities were slower than matched controls with minor orthopedic injuries or than non-injured controls, respectively, in responding to traffic hazards as presented in an experimental video task (35, 36).

In contrast, a recent meta-analysis including eight studies published between 1990 and 2015 indicated that there were no significant differences between individuals with TBI and non-injured individuals in the objective risk of motor vehicle collisions (37). This meta-analysis also demonstrated that based on self-reported data, the risk for motor vehicle collisions was surprisingly higher for non-injured individuals. But still, data showed that individuals with TBI performed worse during on-road assessments and had more problems with vehicular control. A previous review comprising selected studies which included at least 100 participants, control groups, and investigated chronic effects (6 months or longer) concluded that TBI did not lead to increased risks for crashes or driving violations (38). However, this review called attention on other issues that may affect driving, such as the propensity for risk-taking behavior (39), anger issues that may result in later driving problems (40), and the role of executive functions in driving (41). The authors suggested the need to include return to driving as an outcome and the importance of studying the effects of risk-taking and anger issues on driving behaviors after TBI. The aforementioned inconsistencies in outcomes may in part be due to varying levels of availability and access to neurorehabilitation and driving evaluation services across study settings. Also, inconsistencies could be explained by methodological differences between studies for data sources (e.g., self-reported vs. official records) and variables such as driving exposure/experience and TBI severity that are not systematically reported.

The current study thus aimed to explore self-reported and objective (data from official driving records) driving behaviors and offenses in a group of: (a) individuals with TBI, 2–3 years after the end of rehabilitation, who had been evaluated for driving ability following their TBI and deemed fit to return to driving (TBI-DE); (b) individuals with TBI, 2–3 years post-rehabilitation, who had continued to drive following their TBI and did not require a driving evaluation (TBI-NE); and (c) non-injured drivers from the general population. The main objectives were to: (a) compare self-reported driving behaviors and road offenses (i.e., driving habits, driving self-efficacy, driving anger expression, driving-related sensation-seeking, and driving violations/errors, as well as the number of road accidents, driving offenses, and demerit points) between the three groups of participants; (b) compare data from official driving records (i.e., number of road accidents, their seriousness, driving offenses, and demerit points) between the three groups of participants; (c) compare pre-injury and post-rehabilitation data from official driving records (i.e., number of accidents, their seriousness, driving offenses, and demerit points between the two TBI groups; and (d) explore the relationship between self-reported driving behaviors, and number of accidents and offenses in driving records of participants with TBI post-rehabilitation.

This study was conducted within a publicly funded and universally accessible TBI continuum of care. When needed, comprehensive person-centered interdisciplinary neurorehabilitation is freely available to individuals with TBI. Furthermore, the driving license of an individual with TBI is systematically suspended when indicated by the medical and rehabilitation staff. Within the continuum of care, individuals with TBI are referred to one of the driving evaluation programs in the rehabilitation centers for an evidence-based formal assessment of driving fitness. As such, this is a rehabilitation context with equality of access and systematic referral for evaluation of driving fitness. Given the context of unequal access to rehabilitation in which most of the previous studies have been conducted, the discordant results could be in part due to differential access to neurorehabilitation and driving evaluation services. Based on this context permeating our hypotheses, we anticipated that the TBI-DE group would mainly comprise individuals with moderate to severe TBI. Considering the previously highlighted long-term post-TBI cognitive and behavioral issues affecting driving that often persist even following neurorehabilitation, we hypothesized that the TBI-DE group, but not the TBI-NE group, would present more objective road accidents and offenses in the last 2 years (i.e., post-rehabilitation) compared to non-injured controls, but that their self-reported driving behaviors (i.e., driving self-efficacy, anger expression, sensation-seeking, violations/errors) would be similar to those of the other two groups. Given the discordant findings in the literature, we did not anticipate any hypotheses for objectives c and d given their exploratory nature.

## Methods

### Participants

A total of 162 participants took part in this study, including a convenience sample of 72 adults with TBI, and 90 non-injured controls. Participants with TBI were recruited from TBI rehabilitation programs in seven different neurorehabilitation centers across Quebec, Canada. The TBI-DE group consisted of 48 adults whose license had been suspended after the injury (based on a medical decision while in hospital or during rehabilitation) and reinstated following a driving evaluation during outpatient rehabilitation. The TBI-NE group included 24 adults with TBI who had maintained their driving privileges without undergoing a driving evaluation. Non-injured controls included 90 adults without any diagnosed neurological or psychiatric disorder.

Participants with TBI were recruited based on the following inclusion criteria: (a) adults having received a diagnosis of TBI, according to the TBI guidelines put forward by the Quebec Ministry of Health (42), with a mild (Glasgow Coma Scale—GCS score 13–15), moderate (GCS score 9–12) or severe TBI (GCS score 3–8); (b) 18 to 60 years of age; (c) having participated in an interdisciplinary comprehensive rehabilitation program following their TBI; (d) between 2 and 3 years post-TBI rehabilitation; and (e) possession of a valid driver's license. Non-injured controls were recruited based on the following inclusion criteria: (a) 18–60 years of age; (b) self-report of an absence of any diagnosed neurological or psychiatric disorder; and (c) possession of a valid driver's license. All participants reported driving an automobile more than 1,000 km/year in the previous 2 years, as well as in the 2 years predating the injury for the TBI groups. The demographic characteristics and injury-related variables for the three groups are reported in Table 1.

**Table 1:** Demographic, injury-related characteristics, and driving habits for the TBI-DE, TBI-NE, and non-injured control groups.

**Variables**	**TBI-DE** ***(n=48)***		**TBI-NE (*****n*** **=** **24)**		**Non-injured controls (*****n*** **=** **90)**	
	***N* (%)**	***M* (*SD*)**	***N* (%)**	***M* (*SD*)**	***N* (%)**	***M* (*SD*)**
Sex						
Male	35 (73)		12 (50)		54 (40)	
Female	13 (27)		12 (50)		36 (40)	
Age (years)^*^		42.2 (11.5)		36.5 (9.9)		43.8 (11.4)
Injury severity^***^						
Mild TBI	13 (27.1)		18 (75)		–	–
Moderate TBI	11 (22.9)		4 (16.7)		–	–
Severe TBI	24 (50)		2 (8.3)		–	–
Driving experience (months)^**^		300 (135)		199 (94)		301 (123)
Kilometers traveled per year		19 082 (24 827)		14 388 (10 319)		16 319 (18 825)
Preferred route taken						
Less than 10 km	9 (6)		7 (4)		20 (12)	
Between 10 and 50 km	23 (14)		9 (6)		47 (29)	
Between 51 and 100 km	4 (3)		1 (1)		7 (4)	
More than 100 km	1 (1)		4 (3)		2 (2)	
Variable distances	11 (7)		3 (2)		14 (9)	
Access to *SAAQ* Records^***^						
Yes	30 (62.5)		19 (79.2)		22 (24.4)	
No	18 (37.5)		5 (20.8)		68 (75.6)	

*SAAQ, Automobile Insurance Board of Quebec (Société d'Assurance Automobile du Québec)*.

* p < 0.05,

** p < 0.01,

*** *p < 0.001*.

### Procedure

The Research Ethics Board (REB) of the Center for Interdisciplinary Research in Rehabilitation of Greater Montreal of the *CIUSSS du Centre-Sud-de-l'Île-de-Montréal (CIUSSS CSMTL)* approved the current study. Recruitment took place between September 2013 and March 2016. Participants with TBI were recruited from seven rehabilitation centers in the province of Quebec providing driving evaluation programs for individuals with motor, perceptual, or cognitive disabilities. These programs offer fitness to drive assessments, driving skills training, and vehicle adaptation services to individuals referred from other programs within the rehabilitation centers or from outside sources. Research coordinators from the rehabilitation centers contacted potential participants with TBI (TBI-DE and TBI-NE groups) who had finished their rehabilitation program between September 2010 and March 2013 (i.e., 2–3 years post-rehabilitation) and invited them to participate in the study. Non-injured controls from the community were recruited by the professional external interview agency that conducted the interviews. A financial compensation of 15$CAD was provided to all participants.

Data were gathered using a semi-structured telephone interview conceived by the research team, records from the Automobile Insurance Board of Quebec (*Société d'Assurance Automobile du Québec-SAAQ*), and medical records. Participants provided informed consent to participate in the study and could also accept or deny access to their driving records. Access to medical records of participants with TBI also required informed consent. Telephone interviews were conducted by a professional external interview agency (e.g., 30 min approximately, using verbatims provided by the research team). During the telephone interview, non-injured controls and participants with TBI provided information (i.e., self-report) for the previous 2 years (i.e., post-rehabilitation for the TBI groups) regarding their driving habits (i.e., driving experience in months, number of kilometers traveled per year, and most frequent type of route taken in terms of traveling distances), as well as the number of road accidents, number of driving offenses, and demerit points. Objective data for road accidents, the seriousness of accidents (i.e., minor collisions vs. accidents resulting in bodily harm or death, as defined by the *SAAQ* regulatory body), driving offenses and demerit points were also obtained from participants' official driving records for the previous 2 years for all participants (i.e., 2–3 years post-rehabilitation for the TBI groups), as well as for the 2 years preceding injury in the TBI groups. In the province of Quebec, demerit point brackets are determined on the basis of the type of license, where a four-point bracket applies to holders of a learner's license or those who have held their license for fewer than 5 years, and 8-, 12-, and 15-point bracket apply, respectively to driver's license holders under age 23, aged 23 or 24, or aged 25 or older (*SAAQ*, https://saaq.gouv.qc.ca/en/drivers-licences/demerit-points/). During the telephone interview, self-reported driving behaviors were measured for all participants using the following outcome measures.

### Measures

#### Driving Self-Efficacy Scale (DSES)

The DSES is a self-report 12-item questionnaire to estimate the perception of driving abilities (43). Using a 7-point Likert scale (ranging from 1 = “Certainly so” to 7 = “Certainly not”), respondents indicate their agreement (e.g., “Driving a car is easy”). Three items are reverse-scored (i.e., 10, 11, and 12). Means of self-ratings across items are calculated; higher scores indicate better driving self-efficacy. The DSES has shown good internal consistency for the original English version (Cronbach's α = 0.92) (43) and the French version (Cronbach's α = 0.88) (44). Bourrat et al. (45) adapted the French version to individuals with brain injury (Cronbach's α = 0.87).

#### Driving Anger Expression Inventory (DAX)

The DAX is a 49-item questionnaire used to estimate the expression of anger on the road including constructive coping and expression of anger while driving (46). Respondents are asked to rate the frequency of specific reactions while driving in a four-point scale (i.e., 1 = “Almost never” to 4 = “Almost always”). The DAX captures four different dimensions: (a) verbal aggressive expression (12 items; e.g., “Swear at the driver aloud”), (b) use of the vehicle to express anger (11 items; e.g., “Do to drivers what they did to me”), (c) personal physical aggressive expression of anger (11 items; e.g., “Try to get out and have a physical fight”), and (d) adaptive/constructive expression of anger (15 items; e.g., “Accept there are frustrating situations”). The DAX has shown evidence of validity and reliability with internal consistency for its subscales (Cronbach's α ranging from 0.84 to 0.89) (47). Mean of self-ratings across items are calculated for each subscale; higher scores indicate increased levels of anger expression (i.e., subscales a, b, and c) or the use of a more adaptive expression of anger (i.e., subscale d). For this study, we used the French version of the DAX that includes three dimensions: (a) verbal aggressive expression, (b) use of the vehicle to express anger, and (c) adaptive/constructive expression of anger (48, 49). Factor analysis of the French version of the DAX supports the removal of the dimension about the personal physical aggressive expression of anger, with acceptable to satisfactory internal consistencies for the remaining dimensions (Cronbach's α between 0.64 and 0.83) (48, 49).

#### Driving-Related Sensation-Seeking Questionnaire (DRSS)

The DRSS is a 7-item self-report questionnaire that assesses physical and social risk-taking while driving (50). The DRSS has been adapted to French (51). Internal consistencies have been established for both the English (Cronbach's α = 0.84) (50) and the French version (Cronbach's α = 0.68) (51). Respondents are asked to rate each item on a 5-point scale ranging from 1 (i.e., “not true at all”) to 5 (i.e., “absolutely true”) (e.g., “I often feel like being a racing driver”; “I would like to learn how to drive cars that can go faster than 300 km/h”). Mean scores of self-ratings across items are calculated and higher mean scores indicate more driving-related sensation-seeking.

#### Driving Behavior Questionnaire (DBQ)

The driving behavior questionnaire is a self-report 12-item questionnaire to evaluate behaviors related to driving violations and errors (52, 53). Respondents rate the frequency of each behavior using a 6-point Likert scale ranging from 0 (i.e., “Never”) to 5 (i.e., “Nearly all the time”). The DBQ captures three different dimensions: (a) fast driving (5-items; e.g., “Speeding on a residential road”), (b) maintaining progress (4-items; e.g., “Jumping lights”), and (c) anger/hostility (3-items; e.g., “Sounding horn”). Mean scores of self-ratings across items are calculated and higher scores suggest more driving violations and errors while driving. It has been adapted to French (11-item, 4-point Likert scale) from its original format with very good reliability (Cronbach's α = 0.86) (48).

### Statistical Analyses

Statistical analyses were conducted with IBM SPSS® version 25 (54). Descriptive statistics (means, standard deviations, percentages) were calculated for the variables of interest. One-way between-groups analyses of variance and Tukey HSD *post-hoc* tests or independent-samples *t*-tests were conducted to compare participants' characteristics, driving habits (e.g., age, driving experience in months, and kilometers traveled per year), and driving behaviors (e.g., self-reported driving self-efficacy, driving anger expression, driving-related sensation-seeking, driving violations/errors, as well as self-reported, and official records of number of road accidents, driving offenses, and demerit points). Chi-square or Fisher's exact tests were computed to evaluate differences in sex, TBI severity, type of route taken, and seriousness of accidents (i.e., minor collisions or accidents resulting in bodily harm or death) between the three groups. Paired-samples *t*-tests were calculated to compare pre-injury and post-rehabilitation official records of number of accidents, driving offenses, and demerit points in participants with TBI. Pearson product-moment correlation coefficients were calculated to examine, for the past 2 years in participants with TBI (i.e., post-rehabilitation), the relationships between self-reported driving behaviors, and objective number of accidents and driving offenses. Correlation coefficients were interpreted using the following guidelines for the behavioral sciences (55): (a) small (*r* = 0.1–0.29), (b) medium (*r* = 0.3–0.49), and (c) large (*r* = 0.5–1). Statistical significance was set at an alpha level of 0.05.

## Results

### Participants' Characteristics and Driving Habits

As indicated in Table 1, there were no statistically significant differences regarding sex between the three groups. There was a statistically significant difference in age, *F*_(2, 159)_ = 4.01, *p* = 0.02. The effect size (eta squared) was small, at 0.04. *Post-hoc* comparisons indicated that the TBI-NE group was younger than the control group. There were no statistically significant differences in age between the TBI groups or between the TBI-DE group and the control group. Regarding injury severity, the TBI-DE group had more participants with moderate and severe injuries (72.9%) than the TBI-NE group, which comprised mainly individuals with mild injuries (75%), *X*^2^ (2, *n* = 72) = 16.52, *p* = 0.0003.

In terms of driving habits, there was a statistically significant difference in months of driving experience between the three groups, *F*_(2, 159)_ = 6.98, *p* = 0.001. The effect size (eta squared) was medium, at 0.08. *Post-hoc* comparisons indicated that the TBI-NE group had significantly fewer months of driving experience as compared to the TBI-DE group and the control group. There were no significant differences between the TBI-DE and the control group in months of driving experience. There were no significant group differences in kilometers traveled per year. Preferred traveling distances in terms of routes taken were also similar in the three groups, with distances between 10 and 50 km being the most frequently driven. Compared to participants with TBI, the non-injured control group was less likely to authorize access to their driving records, *X*^2^ (2, *n* = 162) = 32.71, *p* = 0.00001.

### Self-Reported Driving Behaviors

As shown in Table 2, the mean scores for the groups with TBI (TBI-DE and TBI-NE) and the non-injured controls did not differ significantly in terms of self-reported behaviors related to driving self-efficacy (DSES), driving-related sensation-seeking (DRSS), or the use of a vehicle to express anger (DAX subscale). On the contrary, there was a statistically significant main effect, with a small effect size just below the medium range, for verbal aggressive expression of anger (DAX subscale). *Post-hoc* comparisons indicated significantly lower mean scores for verbal aggressive expression of anger in the TBI-DE group compared to the control group. Mean scores for the verbal aggressive expression of anger were comparable between the TBI-DE and the TBI-NE groups, and between the TBI-NE and the control group. In addition, there was a statistically significant main effect, with a small effect size, for driving violations/errors (DBQ). *Post-hoc* tests showed that the TBI-DE group reported significantly fewer driving violations/errors than the TBI-NE group, but there were no statistically significant differences between each of the TBI groups and non-injured controls.

**Table 2:** Means, standard deviations, and analyses of variance for self-reported driving behaviors in the TBI-DE, TBI-NE, and non-injured control groups.

Self-reported driving behaviors^*^	**TBI-DE (*****n*** **=** **48)**		**TBI-NE (*****n*** **=** **24)**		**Non-injured controls (*****n*** **=** **90)**		***F*_**(2, 159)**_**	***p***	*****η^2^*****
	***M***	***SD***	***M***	***SD***	***M***	***SD***			
Driving self-efficacy (DSES)	3.78	0.29	3.82	0.29	3.86	0.31	1.25	0.29	0.02
Driving anger expression inventory (DAX)									
Verbal aggressive expression of anger	1.40	0.39	1.52	0.36	1.60	0.41	4.06	0.02	0.05
Use of the vehicle to express anger	1.11	0.15	1.12	0.14	1.15	0.16	1.25	0.29	0.02
Adaptive/constructive expression of anger	2.89	0.69	2.86	0.53	2.85	0.61	0.05	0.95	0.00
Driving-Related	1.86	0.46	2.14	0.85	2.04	0.55	2.28	0.10	0.03
Sensation-seeking (DRSS)									
Driving behavior questionnaire (DBQ)	1.26	0.24	1.43	0.42	1.33	0.24	3.14	0.04	0.04

* *Self-reported driving behaviors at the time of the study (i.e., 2–3 years post-rehabilitation for TBI groups)*.

### Self-Reported and Objective Road Accidents, Offenses, and Demerit Points

As shown in Table 3, the three groups did not differ in terms of self-reported number of accidents, driving offenses or the number of demerit points for the past 2 years (i.e., post-rehabilitation for TBI groups). However, based on objective driving records, there was a significant main effect, with a medium-almost large effect size, for the number of demerit points in the last 2 years. *Post-hoc* analyses indicated that the TBI-DE group had significantly more demerit points compared to non-injured controls. The TBI-DE group also showed a tendency, with a medium effect size, toward more driving offenses in the past 2 years, but this difference did not reach statistical significance. There were no significant differences between the TBI groups in pre-injury number of accidents, driving offenses or demerit points documented in driving records.

**Table 3:** Means, standard deviations, *t*-tests, and analyses of variance for self-reported and objective road accidents, offenses, and demerit points in the TBI-DE, TBI-NE, and non-injured control groups.

**Driving accidents, offenses and demerit points**	**TBI-DE (*****n*** **=** **48)**		**TBI-NE (*****n*** **=** **24)**		**Non-injured controls (*****n*** **=** **90)**		***F*_**(2, 159)**_**	***p***	*****η^2^*****
	***M***	***SD***	***M***	***SD***	***M***	***SD***			
SELF-REPORT PAST 2 YEARS^*^									
Number of accidents	0.31	0.47	0.21	0.41	0.23	0.43	0.66	0.52	0.008
Number of driving offenses	1.71	0.91	1.56	1.33	1.40	0.71	0.55	0.58	0.02
Demerit points	1.32	2.30	1.22	2.41	0.88	1.87	0.77	0.46	0.009
	**TBI-DE (*****N*** **=** **30)**		**TBI-NE (*****N*** **=** **19)**		**Non-injured controls (*****N*** **=** **22)**		***F***_**(2, 68)**_ **or** ***t***_**(47)**_	***p***	***η^2^*** **or Cohen's** ***d***
	***M***	***SD***	***M***	***SD***	***M***	***SD***			
**OFFICIAL DRIVING RECORD**									
Number of accidents past 2 years^*^	0.37	0.56	0.26	0.65	0.18	0.40	0.75	0.47	0.02
Number of accidents pre-injury^**^	0.20	0.48	0.16	0.50	–	–	0.28	0.78	0.08
Number of driving offenses past 2 years^*^	1.13	1.48	0.79	0.92	0.41	0.80	2.47	0.09	0.06
Number of driving offenses pre-injury^**^	0.67	1.12	0.47	0.84	–	–	0.67	0.51	0.20
Demerit points past 2 years^*^	3.17	4.26	2.63	2.63	0.82	1.59	3.54	0.03	0.10
Demerit points pre-injury^**^	0.93	2.05	1.00	2.19	–	–	0.11	0.91	0.03

* *Self-reported and objective accidents and offenses in the 2 years preceding the study, i.e., 2–3 years post-rehabilitation for TBI groups*.

** *Objective driving accidents and offenses in the 2 years preceding injury for TBI groups*.

Fisher exact tests showed a strong significant between-group effect for the seriousness of accidents documented in driving records post-rehabilitation (*p* = 0.0003, two-tailed). The TBI-DE group had a significantly higher frequency of serious accidents resulting in bodily harm or death in the last 2 years (10 serious accidents with two minor accidents), compared to the TBI-NE group (one serious accident with four minor accidents) and the non-injured control group (no serious accidents with four minor accidents). As for differences in pre-injury seriousness of accidents between the TBI groups as documented in driving records, there was a tendency for a higher frequency of serious accidents in the TBI-DE group (four serious accidents without minor accidents) compared to the TBI-NE group (no serious accidents with two minor accidents) (*p* = 0.06, two-tailed).

Comparison between pre-injury and post-rehabilitation objective data from driving records revealed that for both the TBI-DE, *t*_58_ = 2.59, *p* = 0.01, and TBI-NE groups, *t*_36_ = 2.08, *p* = 0.04, the number of demerit points was significantly higher post-rehabilitation than before the injury with a medium-almost large effect size, (both Cohen's *d* values 0.67). There were no statistically significant differences between the pre-injury and post-rehabilitation number of accidents or driving offenses for the TBI-DE and TBI-NE groups.

### Relationships Between Post-rehabilitation Self-Reported Driving Behaviors, and Objective Accidents and Offenses in Participants With TBI

Pearson product-moment correlation coefficients were calculated between post-TBI rehabilitation (i.e. last 2 years) objective accidents and offenses, and self-reported driving behaviors. In the TBI-DE group, there was a medium, negative association between the level of verbal aggressive expression of anger (DAX subscale) and the objective number of accidents (*r* = −0.39; *p* < 0.05). Conversely, the TBI-NE group showed strong positive relationships between the level of verbal aggressive expression of anger and the number of accidents (*r* = 0.54; *p* < 0.05), as well as driving offenses (*r* = 0.47; *p* < 0.05). The TBI-NE group also showed a medium, positive relationship between the level of driving-related sensation-seeking (DRSS) and the number of driving offenses (*r* = 0.46; *p* < 0.05), as well as a strong negative association between the level of adaptive/constructive expression of anger (DAX subscale) and the number of accidents (*r* = −0.6; *p* < 0.01). There were no statistically significant associations in the TBI-DE group. There were no significant correlations for self-reported driving behaviors with age, injury severity, or driving experience.

## Discussion

The current study explored self-reported and objective driving behaviors and offenses in individuals with TBI, 2–3 years post-rehabilitation, having (TBI-DE) or not having undergone a driving evaluation (TBI-NE), with non-injured drivers from the general population. To our knowledge, this is the first multicenter study comparing self-reported and objective driving behaviors in individuals with TBI with or without a driving evaluation, 2–3 years post-rehabilitation, with a non-injured control group. Results show that compared to the TBI-NE and control groups, the TBI-DE group (which comprised mostly individuals with moderate or severe TBI) showed lower or similar self-reported anger- and error-related driving behaviors 2–3 years post-TBI rehabilitation. In contrast, their official driving records (but not their self-report) indicated the presence of a higher number of demerit points and serious accidents. These findings, which have potentially significant public health implications, are generally in line with our hypotheses, although it was not anticipated that the TBI-DE group would report significantly less driving anger expression or errors in driving behaviors. As expected, the TBI-DE group was comparable to the other groups in terms of self-reported driving self-efficacy, driving-related sensation-seeking, and the use of the vehicle to express anger. Of note, there were no group differences in the number of kilometers driven per year and usual traveling distances, indicating that driving habits were similar in the three groups.

Our results suggest that individuals with TBI, in particular those with moderate to severe TBI that have undergone a driving evaluation to get their drivers' licenses reinstated after the injury (i.e., TBI-DE group), may overestimate their driving abilities even though they present more serious accidents and demerit points as documented in driving records. This is further supported by the negative association between self-reported verbal aggressive anger expression and the number of documented accidents post-rehabilitation in this group. That is, participants in the TBI-DE group who reported lower levels of verbal aggressive expression of anger appear to be those who presented more serious accidents. These findings are compatible with a study comparing self-report and motor vehicle records in 47 individuals with TBI who successfully completed a comprehensive driving evaluation and 22 healthy controls (10). All participants self-rated themselves as having excellent or nearly excellent driving skills, indicating that the TBI group had strong confidence in their driving skills at 2.1 years following a driving evaluation. The results of the current study are also in line with another report addressing the cognitive and personality determinants of post-injury driving fitness in 178 individuals with TBI and stroke, where a measure of sensation-seeking turned out to be unrelated to fitness to drive (56). In their study, cognitive ability measures were more important in predicting fitness to drive than driving-related personality traits in individuals with TBI and stroke. Thus, it is not surprising that in the present study driving-related sensation-seeking was not different in participants with TBI and healthy controls.

The presence of more serious road accidents 2–3 years post-rehabilitation in the TBI-DE group is compatible with the results of a study conducted in individuals with severe TBI who resumed driving and presented twice the risk of causing road traffic accidents as compared to pre-injury levels (32). To our knowledge, only the Schultheis et al. (10) study previously examined driving behaviors using a similar methodology (telephone questionnaires and motor vehicle records) including both individuals with TBI having completed a comprehensive, multilevel driver evaluation program, and healthy controls in the United States. Although the authors did not investigate individuals with TBI having received rehabilitation including or not a driving evaluation, our findings are in the same direction of their study. They found that even if the difference was not statistically significant, individuals with TBI were 1.5 times more likely to report being involved in one or more unreported accidents (i.e., minor accidents that did not involve police or insurance documentation) than healthy controls. On the contrary, participants in the control group (*n* = 12, 54.5%) reported taking part in significantly more unsafe driving situations than the group of individuals with TBI (*n* = 8, 20%). This difference can possibly be explained by the fact that their sample of individuals with TBI reported driving less than the sample of healthy controls, and since they measured only minor accidents. Another study showed that drivers having been assessed for driving fitness generally reported modifying their driving behaviors and did not report more crashes compared to pre-injury (15). However, the authors did not compare self-reported data to official driving records. In our study, with similar driving habits (i.e., kilometers driven per year and traveling distances) across groups and separating those who underwent a driving evaluation from those who did not (i.e., TBI-DE and TBI-NE groups, respectively), participants with TBI had a similar post-rehabilitation number of objective minor collisions as controls, but the group having undergone a driving evaluation (comprising 73% of participants with severe and moderate TBI) had more accidents causing death or bodily harm.

Correlational patterns between self-reported driving behaviors and the number of accidents post-rehabilitation were different for the two groups of participants with TBI. Contrary to the TBI-DE group, the group of participants with TBI who did not require a driving evaluation showed positive associations between the aggressive expression of anger or sensation-seeking and the number of accidents and/or driving offenses. Furthermore, this group showed a negative association between self-reported adaptive/constructive expression of anger and the number of accidents post-rehabilitation. Such associations were not present in the group of participants with TBI having passed a driving evaluation. Research conducted with non-injured individuals has suggested similar trends. For example, a study conducted in drivers who did and did not acknowledge problems with driving anger demonstrated that compared to low anger drivers in both groups, high anger drivers engaged in more aggressive and risky behavior on the road and experienced more accident-related outcomes (57). However, it should be underscored that comparisons between pre-injury and post-rehabilitation data from driving records indicated that both TBI groups showed a significantly higher number of demerit points 2–3 years post-rehabilitation. This could suggest that compared to pre-injury levels, the TBI-NE group also shows a high risk for driving offenses leading to demerit points post-rehabilitation. This finding has significant clinical impacts with respect to insuring, during rehabilitation, optimal screening procedures for potentially risky drivers following TBIs of all severities.

Interestingly, even though the number of pre-injury self-reported and objective accidents, offenses and demerit points was similar in the two groups of participants with TBI, the TBI-DE group showed a pre-injury tendency toward more serious accidents. This may be an indication that some individuals in this group were already more at risk for road accidents before their TBI. Hence, even before the injury they could have evaluated themselves as better drivers than they really were, and in turn have a higher predisposition to suffer a (possibly more severe) TBI during a motor vehicle accident. Although this was not an objective of the present study, future research should specifically study the relationships between mechanism of injury and TBI severity, and pre-post TBI driving behaviors as well as road accident/offense history. The literature does suggest that certain groups are more vulnerable to the risk associated with driving. For instance, under different driving conditions, there is a dramatic increase in driving risk among adolescents in the transition period to independent driving (58). Males (59), college students (60), veterans and older adults (61), and individuals with low socioeconomic status or being part of racial/ethnic minorities (62) are at risk for poorer road safety outcomes. Future studies with larger sample sizes could investigate such associations to provide more insight into the relationships between pre-injury and post-rehabilitation driving risks and behaviors in individuals with TBI.

In sum, individuals with TBI, even though they have passed a driving evaluation, may represent a subgroup that is at risk in driving situations since they rated their driving behaviors as being similar to, or better than, non-injured controls even though they presented more serious accidents and demerit points in their driving records than healthy controls in the community. Furthermore, individuals with TBI who have not been identified as needing a driving evaluation during rehabilitation (e.g., individuals with mild or moderate TBI) may also be at risk for increased road offenses resulting in demerit points in the years following rehabilitation. These original findings bring driving safety following a TBI to the forefront in terms of public health and warrant more systematic processes for insuring safe driving following TBI rehabilitation. Problems with self-awareness and executive functions, often seen in more moderate to severe TBIs (but also present in milder TBIs), could be at the basis of these findings, as may be post-TBI memory problems in general (29, 32, 41, 63). Memory problems could also explain between-group differences for self-reported data when compared to official driving records for the number of accidents, driving offenses, and demerit points.

### Limitations and Future Directions

Limitations of this study include self-selection and self-reporting bias, the characteristics of the sample, and the research design. Self-selection bias is inherent to these types of studies. Participants who volunteer for studies on driving may generally find themselves safer to drive (although they may not be aware of their risky driving behaviors). In some cases, individuals may decline participation out of fear that their driving license will be suspended or because they are uncomfortable with the study objectives (3). That could explain, in part, the major trend for non-injured participants to refuse access to their driving records. In the present study, while self-reported driving behaviors were obtained from all participants in the three groups (TBI-DE, TBI-NE, non-injured controls), and the majority of individuals in both TBI groups gave access to their driving records for comparison purposes, a significant proportion of controls did not give such access. This thus warrants caution when interpreting TBI vs. control group differences in objective driving data. Regarding the characteristics of the sample, the TBI-NE group included participants who were younger than the control group, had milder injuries than the TBI-DE group, as expected (this latter difference being inherent to our study objectives comparing groups of individuals with TBI having been referred or not for a driving evaluation), and had less driving experience than the two other groups. However, age, severity, and driving experience did not show a relationship with self-reported driving behaviors neither in the TBI-NE group nor in the TBI-DE group, indicating that they did not appear to influence the results in our study.

For administrative reasons, we were not able to document some characteristics including the cause of the TBI, the exact time between the end of rehabilitation and the completion of the telephone interview (although the interval was limited between 2 and 3 years after the end of rehabilitation for all individuals with TBI), and the date of the driving evaluation (although the driving evaluation is usually conducted within a 1-year interval before the end of rehabilitation). In addition, we did not measure cognitive and psychological functioning or self-awareness simultaneously with self-reported driving behaviors since this was not an objective of our study, but these would all be important aspects to address in future studies. Finally, a cross-sectional study cannot fully capture the temporal evolution of driving behaviors, and longitudinal studies are warranted to track changes in driving behaviors over time following a TBI.

## Conclusions and Recommendations for Practice

In this study participants with TBI having passed a driving evaluation resumed driving at the same level as participants with TBI who did not undergo a driving evaluation and as non-injured controls, but they appeared to underestimate their risky driving behaviors. Self-awareness concerning driving skills should be fully assessed during off- and on-road assessment of driving fitness during rehabilitation (63–66). Close collaboration between driving evaluation professionals and rehabilitation teams should be encouraged to better understand the cognitive, behavioral and psychological/personality characteristics that may impact driving post-TBI in order to specifically target them during interventions, as well as to determine the best time to perform the driving evaluation (67). Furthermore, emphasis should be put on driving retraining (68) even for those individuals with TBI who successfully complete a driving evaluation. Future clinical research should target evaluation of on-road driving evaluation/retraining evidence-based practices, as well as systematic post-rehabilitation follow-up of individuals with TBI who have passed a driving evaluation, as well as those who were not targeted as needing a driving evaluation, but who may be considered at risk.

Based on the results of the current study, the critical review of the literature, and our clinical experience in different rehabilitation fields and more broadly in driving psychology, some clinical and policy recommendations are proposed:

- The finding that compared to pre-injury levels, individuals with TBI had significantly more demerit points post-rehabilitation compared to their pre-injury driving records has important public health implications. We recommend that during rehabilitation individuals with TBI be closely monitored regarding abilities and behaviors related to driving skills. Prevention measures such as mandatory training to increase their driving abilities as well as awareness of risks for road accidents and driving sanctions if they transgress road safety rules, could be systematically implemented even in individuals with TBI who have successfully undergone a driving evaluation process.
- As suggested by Deffenbacher et al. (57), interventions for angry drivers acknowledging that they may have anger-related difficulties could include psychoeducational and psychotherapeutic interventions. On the contrary, interventions for angry drivers who do not accept that they have a problem could include the readiness and motivation to address them and increasing awareness of their problems and risks (e.g., readiness enhancement interventions and motivational interviewing). Those interventions recommended for non-injured individuals could be adapted to individuals with TBI and be part of public policies to prevent negative driving-related outcomes.
- To date, there are no single measures or a combination of measures that will accurately predict who is and who is not a safe driver following a TBI (69). As such, driving evaluation professionals must ensure that they have performed a complete evidence-based assessment of their clients before they proceed to suggest accommodations, driving restrictions, or to stop driving. This should be done in close collaboration with interdisciplinary rehabilitation professionals involved in treating the person with a TBI.
- When available, driving simulators are a controlled and repeatable strategy to measure driver behaviors (70). However, more research is needed to justify their use in clinical practice for assessment and intervention purposes (70–72). But even with normal neuropsychological results, clinicians must be aware that emotional and personality changes can also play a role in driving safety.
- Restricted licensing offers an alternative to license withdrawal in many North American jurisdictions and in Australia to help individuals in the transition to independent and safe driving allowing them to drive only under certain conditions (e.g.,driving in a specific geographical area), but more evidence is needed in the context of TBI (68).

## Author Contributions

MM, PD, and IG designed the study and conducted data collection. AM and MM conducted statistical analyses and drafted the manuscript. All authors reviewed the different iterations of the manuscript and approved the final version.

### Conflict of Interest Statement

The authors declare that the research was conducted in the absence of any commercial or financial relationships that could be construed as a potential conflict of interest.
